# Preexposure Prophylaxis for the Prevention of HIV Infection — 2014 Available Online

**Published:** 2014-05-16

The documents Preexposure Prophylaxis for the Prevention of HIV Infection — 2014: a PHS Clinical Practice Guideline^*^ and a *Clinical Providers’ Supplement*^†^ are now available online.

The guideline and supplement are intended for use by clinicians in the United States providing medical care for persons without human immunodeficiency virus (HIV) infection who are at substantial risk for acquiring it by their sexual or injection drug use behaviors. The guideline is the first federal resource that provides comprehensive, evidence-based information about the provision of daily oral antiretroviral preexposure prophylaxis (PrEP), including how to identify patients with indications for PrEP, guidance for safe prescribing practices, monitoring clinical safety for patients taking PrEP medications, and supporting medication adherence and the reduction of HIV risk behaviors. The supplement provides additional tools and information that might be useful to clinicians prescribing PrEP.

